# 1-(4-Chloro­phen­yl)-4,4,6-trimethyl-3,4-dihydro­pyrimidine-2(1*H*)-thione

**DOI:** 10.1107/S1600536810001777

**Published:** 2010-01-23

**Authors:** Aamer Saeed, Michael Bolte

**Affiliations:** a Department of Chemistry, Quaid-i-Azam University, Islamabad 45320, Pakistan; b Institut für Anorganische Chemie, J. W. Goethe-Universität Frankfurt, Max-von-Laue-Strasse 7, 60438 Frankfurt/Main, Germany

## Abstract

The dihydro­pyrimidine ring of the title compound, C_13_H_15_ClN_2_S, adopts an envelope conformation with five almost coplanar atoms (r.m.s. deviation = 0.054 Å) and the C atom bearing the two methyl substituents deviating from this plane by 0.441 (2) Å. The best plane through the five almost coplanar atoms forms a dihedral angle of 89.56 (5)° with the benzene ring. The crystal packing is characterized by centrosymmetric dimers connected by pairs of N—H⋯S hydrogen bonds.

## Related literature

For details of the biological activity of pyrimidine-2-thio­nes, see: Alam *et al.* (2005 ▶); Sriram *et al.* (2006 ▶); Leite *et al.* (2006 ▶); Kappe (2000 ▶); Rovnyak *et al.* (1995 ▶); Swamy *et al.* (2005 ▶). For a related structure, see: Yamin *et al.* (2005 ▶).
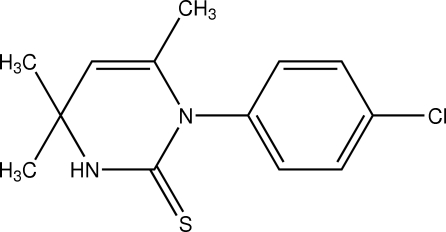


## Experimental

### 

#### Crystal data

C_13_H_15_ClN_2_S
*M* *_r_* = 266.78
Monoclinic, 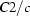

*a* = 20.6710 (18) Å
*b* = 10.8343 (10) Å
*c* = 14.8619 (13) Å
β = 126.026 (5)°
*V* = 2691.9 (4) Å^3^
*Z* = 8
Mo *K*α radiation
μ = 0.42 mm^−1^
*T* = 173 K
0.37 × 0.29 × 0.26 mm

#### Data collection

Stoe IPDS II two-circle diffractometer
Absorption correction: multi-scan (*MULABS*; Spek, 2009 ▶; Blessing, 1995 ▶) *T*_min_ = 0.861, *T*_max_ = 0.899
7624 measured reflections
2512 independent reflections
2134 reflections with *I* > 2σ(*I*)
*R*_int_ = 0.049

#### Refinement

*R*[*F* ^2^ > 2σ(*F* ^2^)] = 0.035
*wR*(*F* ^2^) = 0.093
*S* = 1.02
2512 reflections
161 parameters
H atoms treated by a mixture of independent and constrained refinement
Δρ_max_ = 0.22 e Å^−3^
Δρ_min_ = −0.39 e Å^−3^

### 

Data collection: *X-AREA* (Stoe & Cie, 2001 ▶); cell refinement: *X-AREA*; data reduction: *X-AREA*; program(s) used to solve structure: *SHELXS97* (Sheldrick, 2008 ▶); program(s) used to refine structure: *SHELXL97* (Sheldrick, 2008 ▶); molecular graphics: *XP* (Sheldrick, 2008 ▶); software used to prepare material for publication: *SHELXL97*.

## Supplementary Material

Crystal structure: contains datablocks global, I. DOI: 10.1107/S1600536810001777/zq2028sup1.cif

Structure factors: contains datablocks I. DOI: 10.1107/S1600536810001777/zq2028Isup2.hkl

Additional supplementary materials:  crystallographic information; 3D view; checkCIF report

## Figures and Tables

**Table 1:** Hydrogen-bond geometry (Å, °)

*D*—H⋯*A*	*D*—H	H⋯*A*	*D*⋯*A*	*D*—H⋯*A*
N3—H1⋯S1^i^	0.83 (2)	2.59 (2)	3.4054 (16)	169.1 (17)

Symmetry code: (i) 
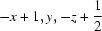
.

**Fig. 1.**
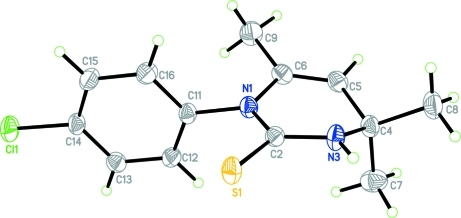
Molecular structure of the title compound. Displacement ellipsoids are drawn at the 50% probability level.

## supplementary crystallographic information

### Comment

The title compound belongs to a novel and rare class of dihydropyrimidine-2-thiones. Their synthesis has been attracting widespread attention due to diverse pharmacological activities such as antibacterial (Alam *et al.*, 2005), antitumour (Swamy *et al.*, 2005), antioxidative (Sriram *et al.*, 2006), analgesic and anti-inflammatory properties (Leite *et al.*, 2006; Kappe, 2000). In addition, these compounds act as antihypertensive agents as well as calcium channel blockers and neuropeptide Y antagonists (Rovnyak *et al.*, 1995). The formation of the closely related 4,4,6-trimethyl-1-phenyl-3,4-dihydropyrimidine-2(1*H*)-thione as a side product during the reaction of cinnamoyl isothiocyanate and aniline to afford the corresponding thiourea derivative has been reported (Yamin *et al.*, 2005) The title compound was prepared by the reaction of 4-chloroaniline with 4-methylpent-3-en-2-one in presence of potassium thiocyanate in acetone.

The dihydropyrimidine ring of the title compound adopts an envelope conformation with five almost coplanar atoms (r.m.s. deviation 0.054 Å) and the carbon atom bearing the two methyl substituents deviating from this plane by 0.441 (2) Å. The best plane through the six ring atoms forms a dihedral angle of 89.42 (5)° with the phenyl ring. The crystal packing is characterized by centrosymmetric dimers connected by N—H···S hydrogen bonds.

### Experimental

Potassium thiocyanate (5.4 mmol) was added to a stirred mixture of 4-methylpent-3-en-2-one (5.4 mmol), 4-chloroaniline (5.4 mmol) in dry acetone. The reaction mixture was refluxed for 3 hours. On completion of the reaction, the reaction mixture was cooled to room temperature and poured into ice-water. The precipitated compound was recrystallized from methanol to afford the title dihydropyrimidine-2-thione (62%). Recrystallization from methanol afforded the title compound as colourless crystals: Anal. calcd. for C_13_H_15_N_2_S: C, 58.53; H, 5.67; N, 10.50; S, 12.02%; found: C, 58.49; H, 5.72; N, 10.61; S, 12.14%;%.

### Refinement

Hydrogen atoms were located in a difference Fourier map but they were all included in calculated positions [C_aromatic_—H = 0.95 Å; C_methyl_—H = 0.98°] and refined as riding [*U*_iso_(H) = 1.2*U*_eq_(C) or *U*_iso_(H) = 1.5*U*_eq_(C_methyl_)]. The methyl groups were allowed to rotate but not to tip.

### Crystal data

**Table:**

C_13_H_15_ClN_2_S	*F*(000) = 1120
*M**_r_* = 266.78	*D*_x_ = 1.317 Mg m^−^^3^
Monoclinic, *C*2/*c*	Mo *K*α radiation, λ = 0.71073 Å
Hall symbol: -C 2yc	Cell parameters from 6686 reflections
*a* = 20.6710 (18) Å	θ = 3.4–26.1°
*b* = 10.8343 (10) Å	µ = 0.42 mm^−^^1^
*c* = 14.8619 (13) Å	*T* = 173 K
β = 126.026 (5)°	Block, colourless
*V* = 2691.9 (4) Å^3^	0.37 × 0.29 × 0.26 mm
*Z* = 8	

### Data collection

**Table:**

Stoe IPDS II two-circle diffractometer	2512 independent reflections
Radiation source: fine-focus sealed tube	2134 reflections with *I* > 2σ(*I*)
graphite	*R*_int_ = 0.049
ω scans	θ_max_ = 25.7°, θ_min_ = 3.4°
Absorption correction: multi-scan (*MULABS*; Spek, 2009; Blessing, 1995)	*h* = −25→24
*T*_min_ = 0.861, *T*_max_ = 0.899	*k* = −13→13
7624 measured reflections	*l* = −16→18

### Refinement

**Table:**

Refinement on *F*^2^	Primary atom site location: structure-invariant direct methods
Least-squares matrix: full	Secondary atom site location: difference Fourier map
*R*[*F*^2^ > 2σ(*F*^2^)] = 0.035	Hydrogen site location: inferred from neighbouring sites
*wR*(*F*^2^) = 0.093	H atoms treated by a mixture of independent and constrained refinement
*S* = 1.02	*w* = 1/[σ^2^(*F*_o_^2^) + (0.0634*P*)^2^] where *P* = (*F*_o_^2^ + 2*F*_c_^2^)/3
2512 reflections	(Δ/σ)_max_ = 0.001
161 parameters	Δρ_max_ = 0.22 e Å^−^^3^
0 restraints	Δρ_min_ = −0.39 e Å^−^^3^

### Special details

**Table:**

Geometry. All e.s.d.'s (except the e.s.d. in the dihedral angle between two l.s. planes) are estimated using the full covariance matrix. The cell e.s.d.'s are taken into account individually in the estimation of e.s.d.'s in distances, angles and torsion angles; correlations between e.s.d.'s in cell parameters are only used when they are defined by crystal symmetry. An approximate (isotropic) treatment of cell e.s.d.'s is used for estimating e.s.d.'s involving l.s. planes.
Refinement. Refinement of *F*^2^ against ALL reflections. The weighted *R*-factor *wR* and goodness of fit *S* are based on *F*^2^, conventional *R*-factors *R* are based on *F*, with *F* set to zero for negative *F*^2^. The threshold expression of *F*^2^ > σ(*F*^2^) is used only for calculating *R*-factors(gt) *etc*. and is not relevant to the choice of reflections for refinement. *R*-factors based on *F*^2^ are statistically about twice as large as those based on *F*, and *R*- factors based on ALL data will be even larger.

### Fractional atomic coordinates and isotropic or equivalent isotropic displacement parameters (Å^2^)

**Table:**

	*x*	*y*	*z*	*U*_iso_*/*U*_eq_	
S1	0.57142 (2)	0.69991 (4)	0.42770 (3)	0.02504 (14)	
Cl1	0.68751 (3)	0.59306 (4)	0.93548 (3)	0.03636 (15)	
N1	0.47205 (7)	0.78438 (12)	0.47357 (11)	0.0219 (3)	
H1	0.4318 (11)	0.7666 (19)	0.2421 (18)	0.027 (5)*	
C2	0.48383 (9)	0.75825 (14)	0.39373 (13)	0.0202 (3)	
N3	0.42364 (8)	0.78513 (13)	0.28874 (12)	0.0239 (3)	
C4	0.33943 (9)	0.80299 (15)	0.24811 (13)	0.0240 (4)	
C5	0.34219 (9)	0.86623 (15)	0.34036 (14)	0.0251 (3)	
H5	0.2983	0.9165	0.3221	0.030*	
C6	0.40392 (9)	0.85420 (15)	0.44661 (14)	0.0238 (3)	
C7	0.29744 (11)	0.67811 (18)	0.21978 (17)	0.0364 (4)	
H7A	0.3015	0.6356	0.1651	0.055*	
H7B	0.2410	0.6907	0.1886	0.055*	
H7C	0.3229	0.6280	0.2874	0.055*	
C8	0.29878 (10)	0.88397 (18)	0.14363 (15)	0.0348 (4)	
H9A	0.3273	0.9627	0.1621	0.052*	
H9B	0.2432	0.8993	0.1157	0.052*	
H9C	0.2999	0.8416	0.0863	0.052*	
C9	0.40992 (11)	0.91451 (18)	0.54228 (15)	0.0342 (4)	
H8A	0.3607	0.9608	0.5143	0.051*	
H8B	0.4557	0.9710	0.5804	0.051*	
H8C	0.4173	0.8511	0.5946	0.051*	
C11	0.52708 (9)	0.73685 (15)	0.58519 (12)	0.0209 (3)	
C12	0.51342 (9)	0.62101 (15)	0.60981 (13)	0.0236 (3)	
H12	0.4698	0.5727	0.5531	0.028*	
C13	0.56377 (9)	0.57500 (15)	0.71809 (14)	0.0251 (3)	
H13	0.5550	0.4955	0.7359	0.030*	
C14	0.62664 (9)	0.64743 (15)	0.79878 (13)	0.0237 (3)	
C15	0.64175 (9)	0.76243 (16)	0.77479 (14)	0.0271 (4)	
H15	0.6859	0.8099	0.8313	0.033*	
C16	0.59136 (10)	0.80791 (15)	0.66666 (14)	0.0260 (4)	
H16	0.6009	0.8869	0.6488	0.031*	

### Atomic displacement parameters (Å^2^)

**Table:**

	*U*^11^	*U*^22^	*U*^33^	*U*^12^	*U*^13^	*U*^23^
S1	0.0186 (2)	0.0362 (3)	0.0192 (2)	0.00399 (14)	0.01047 (17)	0.00264 (15)
Cl1	0.0372 (2)	0.0389 (3)	0.0182 (2)	0.00307 (17)	0.00804 (19)	0.00696 (16)
N1	0.0217 (6)	0.0262 (7)	0.0175 (7)	0.0037 (5)	0.0114 (6)	0.0023 (5)
C2	0.0220 (7)	0.0197 (7)	0.0190 (7)	−0.0011 (6)	0.0120 (6)	0.0013 (6)
N3	0.0193 (6)	0.0360 (8)	0.0162 (7)	0.0046 (5)	0.0104 (6)	0.0030 (5)
C4	0.0182 (7)	0.0276 (8)	0.0213 (8)	0.0039 (6)	0.0089 (7)	0.0023 (6)
C5	0.0230 (7)	0.0254 (8)	0.0273 (8)	0.0045 (6)	0.0151 (7)	0.0027 (6)
C6	0.0252 (7)	0.0233 (8)	0.0264 (8)	0.0024 (6)	0.0172 (7)	0.0022 (6)
C7	0.0299 (9)	0.0330 (9)	0.0442 (11)	−0.0031 (7)	0.0206 (9)	−0.0078 (8)
C8	0.0298 (9)	0.0410 (10)	0.0232 (9)	0.0098 (7)	0.0097 (8)	0.0067 (8)
C9	0.0365 (9)	0.0395 (10)	0.0301 (9)	0.0075 (7)	0.0216 (8)	−0.0014 (8)
C11	0.0222 (7)	0.0256 (8)	0.0157 (7)	0.0024 (6)	0.0116 (6)	0.0015 (6)
C12	0.0222 (7)	0.0234 (8)	0.0223 (8)	−0.0024 (6)	0.0114 (7)	−0.0016 (6)
C13	0.0270 (8)	0.0230 (8)	0.0246 (8)	0.0004 (6)	0.0148 (7)	0.0034 (6)
C14	0.0244 (7)	0.0286 (8)	0.0153 (7)	0.0039 (6)	0.0101 (6)	0.0024 (6)
C15	0.0259 (8)	0.0291 (8)	0.0198 (8)	−0.0044 (6)	0.0097 (7)	−0.0026 (7)
C16	0.0294 (8)	0.0248 (8)	0.0226 (8)	−0.0040 (6)	0.0146 (7)	0.0006 (6)

### Geometric parameters (Å, °)

**Table:**

S1—C2	1.6904 (15)	C8—H9A	0.9800
Cl1—C14	1.7465 (16)	C8—H9B	0.9800
N1—C2	1.374 (2)	C8—H9C	0.9800
N1—C6	1.4327 (19)	C9—H8A	0.9800
N1—C11	1.4462 (19)	C9—H8B	0.9800
C2—N3	1.336 (2)	C9—H8C	0.9800
N3—C4	1.482 (2)	C11—C12	1.382 (2)
N3—H1	0.83 (2)	C11—C16	1.390 (2)
C4—C5	1.504 (2)	C12—C13	1.397 (2)
C4—C7	1.527 (2)	C12—H12	0.9500
C4—C8	1.534 (2)	C13—C14	1.382 (2)
C5—C6	1.330 (2)	C13—H13	0.9500
C5—H5	0.9500	C14—C15	1.381 (2)
C6—C9	1.502 (2)	C15—C16	1.393 (2)
C7—H7A	0.9800	C15—H15	0.9500
C7—H7B	0.9800	C16—H16	0.9500
C7—H7C	0.9800		
			
C2—N1—C6	120.78 (13)	H9A—C8—H9B	109.5
C2—N1—C11	119.87 (12)	C4—C8—H9C	109.5
C6—N1—C11	119.30 (13)	H9A—C8—H9C	109.5
N3—C2—N1	116.59 (13)	H9B—C8—H9C	109.5
N3—C2—S1	121.92 (13)	C6—C9—H8A	109.5
N1—C2—S1	121.46 (11)	C6—C9—H8B	109.5
C2—N3—C4	124.65 (15)	H8A—C9—H8B	109.5
C2—N3—H1	114.6 (14)	C6—C9—H8C	109.5
C4—N3—H1	117.4 (13)	H8A—C9—H8C	109.5
N3—C4—C5	106.41 (13)	H8B—C9—H8C	109.5
N3—C4—C7	109.73 (13)	C12—C11—C16	120.79 (14)
C5—C4—C7	111.46 (15)	C12—C11—N1	118.79 (13)
N3—C4—C8	107.47 (14)	C16—C11—N1	120.40 (14)
C5—C4—C8	111.52 (14)	C11—C12—C13	119.95 (14)
C7—C4—C8	110.09 (14)	C11—C12—H12	120.0
C6—C5—C4	122.23 (14)	C13—C12—H12	120.0
C6—C5—H5	118.9	C14—C13—C12	118.71 (15)
C4—C5—H5	118.9	C14—C13—H13	120.6
C5—C6—N1	118.87 (15)	C12—C13—H13	120.6
C5—C6—C9	124.65 (15)	C15—C14—C13	121.88 (14)
N1—C6—C9	116.40 (13)	C15—C14—Cl1	119.14 (12)
C4—C7—H7A	109.5	C13—C14—Cl1	118.97 (13)
C4—C7—H7B	109.5	C14—C15—C16	119.19 (14)
H7A—C7—H7B	109.5	C14—C15—H15	120.4
C4—C7—H7C	109.5	C16—C15—H15	120.4
H7A—C7—H7C	109.5	C11—C16—C15	119.47 (15)
H7B—C7—H7C	109.5	C11—C16—H16	120.3
C4—C8—H9A	109.5	C15—C16—H16	120.3
C4—C8—H9B	109.5		
			
C6—N1—C2—N3	−9.4 (2)	C2—N1—C6—C9	−159.70 (15)
C11—N1—C2—N3	168.04 (14)	C11—N1—C6—C9	22.8 (2)
C6—N1—C2—S1	168.43 (11)	C2—N1—C11—C12	−87.68 (19)
C11—N1—C2—S1	−14.1 (2)	C6—N1—C11—C12	89.81 (18)
N1—C2—N3—C4	−19.9 (2)	C2—N1—C11—C16	93.85 (19)
S1—C2—N3—C4	162.28 (12)	C6—N1—C11—C16	−88.66 (19)
C2—N3—C4—C5	36.3 (2)	C16—C11—C12—C13	1.1 (2)
C2—N3—C4—C7	−84.4 (2)	N1—C11—C12—C13	−177.39 (14)
C2—N3—C4—C8	155.86 (16)	C11—C12—C13—C14	0.1 (2)
N3—C4—C5—C6	−26.8 (2)	C12—C13—C14—C15	−1.2 (3)
C7—C4—C5—C6	92.80 (19)	C12—C13—C14—Cl1	177.54 (13)
C8—C4—C5—C6	−143.71 (17)	C13—C14—C15—C16	1.2 (3)
C4—C5—C6—N1	3.6 (2)	Cl1—C14—C15—C16	−177.54 (13)
C4—C5—C6—C9	−179.71 (16)	C12—C11—C16—C15	−1.1 (3)
C2—N1—C6—C5	17.2 (2)	N1—C11—C16—C15	177.36 (15)
C11—N1—C6—C5	−160.24 (15)	C14—C15—C16—C11	−0.1 (3)

### Hydrogen-bond geometry (Å, °)

**Table:**

*D*—H···*A*	*D*—H	H···*A*	*D*···*A*	*D*—H···*A*
N3—H1···S1^i^	0.83 (2)	2.59 (2)	3.4054 (16)	169.1 (17)

Symmetry codes: (i) −*x*+1, *y*, −*z*+1/2.
